# The association between depressive symptoms and insulin resistance, inflammation and adiposity in men and women

**DOI:** 10.1371/journal.pone.0187448

**Published:** 2017-11-30

**Authors:** M’Balu Webb, Melanie Davies, Nuzhat Ashra, Danielle Bodicoat, Emer Brady, David Webb, Calum Moulton, Khalida Ismail, Kamlesh Khunti

**Affiliations:** 1 National Institute for Health Research Biomedical Research Centre—Leicester, University Hospitals of Leicester, Leicester, England; 2 The Leicester Diabetes Centre, University Hospitals of Leicester, Leicester General Hospital, Leicester, England; 3 Diabetes Research Centre, University of Leicester, Leicester General Hospital, Leicester, England; 4 Department of Psychological Medicine, Weston Education Centre, Kings College London, London, England; Vanderbilt University, UNITED STATES

## Abstract

**Introduction:**

Depression has been shown to be associated with elevated leptin levels, low-grade inflammation and insulin resistance. These derangements are often measured in mixed gender cohorts despite the different body compositions and hormonal environments of men and women and gender-specific prevalence and responses to depression.

**Methods:**

A cross-sectional analysis was carried out on a cohort of 639 participants from the ADDITION-Leicester dataset to assess differences in markers of diabetes risk, cardiovascular risk and inflammation in depressed and non-depressed individuals. Depressive symptoms were determined using the WHO (Five) well-being index. Multivariate linear and logistic regression analyses were adjusted for age, sex, ethnicity, body mass index, smoking, social deprivation and activity levels for continuous and binary variables respectively. Further analysis included stratifying the data by gender as well as assessing the interaction between depression and gender by including an interaction term in the model.

**Results:**

Women with depressive symptoms had a 5.3% larger waist circumference (p = 0.003), 28.7% higher HOMA IR levels (p = 0.026), 6.6% higher log-leptin levels (p = 0.01) and 22.37% higher TNF-α levels (p = 0.015) compared with women without. Conversely, depressive symptoms in men were associated with 7.8% lower body fat % (p = 0.015) but 48.7% higher CRP levels (p = 0.031) compared to men without. However, interaction analysis failed to show a significant difference between men and women.

**Conclusions:**

Depressive symptoms are associated with metabolic derangements. Whilst women tended to show elevations in biomarkers related to an increased risk of type 2 diabetes (HOMA IR, leptin and TNF-α), men showed a marked increase in the cardiovascular disease risk biomarker CRP. However, perhaps due to the cohort size, interaction analysis did not show a significant gender difference.

## 1. Introduction

Depression is associated with a significantly increased risk of developing type 2 diabetes (T2DM) and cardiovascular disease (CVD)[1–3]. A meta-analysis by Mezuk and colleagues (2008) estimated a 60% increased risk of diabetes [4], whilst Gan et al (2014) estimated a 30% increased pooled risk for coronary heart disease (CHD) [5]. Depression is also independently associated with increased risk of mortality [6, 7]. The adverse effects of depression on diabetes and cardiovascular outcomes have been attributed to poor lifestyle behaviours including: increased caloric intake and reduced rates of exercise [8, 9]. However, even when these factors have been controlled for, the association between depression and increased risk of CVD and T2DM has persisted [1, 5].

The causal pathways linking depression with metabolic dysregulation have not been fully elucidated, however elevations in insulin resistance (IR), low grade inflammation and leptin have been repeatedly reported [1, 10–13]. More severe cases of depression have been associated with activation of the hypothalamic–pituitary–adrenocortical (HPA) axis and sympathetic nervous system which is known to lead to an increased release of catecholamines which in turn inhibit insulin-induced uptake of glucose in adipocytes [14–17]. However, this affect is relatively modest, for instance, a meta-analysis by Kan et al 2013, detected a small increased risk of IR (d = 0.19 (95% CI: 0.11–0.27)[1]. Cross-sectional studies also show a positive association between depression and levels of proinflammatory cytokines but the causal direction cannot be inferred. Whilst the induction of psychological stress has been shown increase TNF and IL-6, the reverse has also been observed and high baseline levels and the administration of proinflammatory cytokines have been shown to precede new onset of depressive symptoms in prospective studies and clinical trials respectively [18–22]. A further marked feature of depression is that it appears to be associated with altered leptin homeostasis [13, 23]. High circulating leptin, a marker of leptin resistance, is independently associated with both IR and CVD [24, 25]. However, the relevance of leptin in depression vs. T2DM and CVD remains unclear due to the fact that divergent reports have associated depression with both hypo and hyperleptinemia [12, 13, 23, 26]. This is further confounded by reports that hyperleptinemia is associated with both enhanced mood and the onset of depression [27, 28]. Therefore, the potential role of leptin and the increased prevalence of T2DM and CVD with depression requires further study.

Most studies analysing depression and metabolic dysfunction have not considered effect modification by gender. This is despite the marked differences in body mass, body composition and hormonal milieu of each gender which in turn affects basal levels of CVD markers (e.g. CRP [29] and clinical measures of T2DM (e.g. HOMA IR [30]). Additionally, the prevalence of depression differs with sex, with Kessler and colleagues (1997) reporting a 21.3% prevalence in women compared with 12.7% in men [31]; although the risk of mortality from major chronic diseases is higher in depressed men than in women [32]. Furthermore, studies have shown different gender specific responses to depression, for example, increased alcohol use is twice as likely in men than in women [33]. Therefore the following study had two aims: firstly to add to the existing evidence that depressive symptoms are linked to markers of T2DM, CVD and inflammation in a healthy population. Furthermore, we hypothesize that these metabolic disturbances will differ with gender.

## 2. Methods

This study is a secondary analysis of baseline data collected during the screening phase of the ADDITION-Leicester study (NCT00318032), an Anglo-Danish-Dutch randomised control trial assessing of the cost effectiveness of population screening and intensive multi-factorial intervention for Type 2 diabetes. Details of this cohort have been published elsewhere [34]. The Addition study was carried out in accordance with the latest version of the Declaration of Helsinki. The study design was approved by the Nottingham Research Ethics Committee, UK and informed consent of the participants was obtained after the nature of the procedures had been fully explained. Baseline data and samples were collected from n = 6749 participants (healthy subjects with no diagnosis of T2DM) between 2004 and 2008. Between 2008 and 2009, n = 987 random samples were assayed for the following biomarkers: leptin, CRP, TNF-α, IL-6, adiponectin, insulin, PGF, resistin and apolipoprotein A1 & B. On the day of blood collection, participants were assessed for wellbeing using the WHO (Five) wellbeing index, scored as a continuous variable with higher values indicating that the participant had experienced a higher level of wellbeing in the 2 weeks preceding the assessment. The possible raw scores were 0 to 25 and a cut-point of ≤13 was utilised to identify depressive symptoms. Whilst the WHO (Five) questionnaire does not represent a clinically applicable screen for depression, it has been shown to be affective in screening for those with a ‘caseness’ for depression. Additionally, using the cut-off specified, WHO (Five) has been shown to have 100% sensitivity and 78% specificity as a screening tool for depression [35]. WHO (Five) also shows acceptable findings for internal consistency with a Cronbach’s coefficient alpha score of 0.84 [36]. Therefore for the purposes of this report participants with a WHO (5) score of ≤ 13 will be categorised as displaying depressive symptoms.

Of the 987 participants with biomarker data, 307 were excluded because they had missing WHO (Five) data. Additionally, a further 41 participants were excluded for: missing ethnicity data (n = 1), social deprivation scores (n = 7), physical activity scores (n = 31) and/or smoking status data (n = 3). Therefore 639 participants were included in the current analysis. An analysis of the n = 346 participants that were not included (principally because they had not completed the WHO (five) questionnaire, n = 307) showed that they were: older, less likely to be White European, less physically active, more likely to have screen detected T2DM and IGR and showed a higher index of multiple deprivation; as such, non-inclusion of these participants may have introduced some sampling bias (S1 Table).

### 2.1 Population

White Europeans between the ages of 40–75 years and Black and Minority Ethnic (BME) participants (predominantly South Asian) between the ages of 25–75 years were included in the study. A lower age cut-off for BME participants was chosen due to the reported higher risk of T2DM and CVD. People with the following pre-existing conditions are excluded (general practice diagnosis and database recorded), T2DM, terminal illnesses with a likely prognosis of less than 12 months, psychiatric illness likely to hinder informed consent, pregnancy and lactation.

### 2.2 Anthropometric and clinical assessments

Baseline demographic data captured at screening included: age, sex, ethnicity, BMI, smoking, index of social deprivation and activity levels. Ethnicity status was self-assigned using UK population census categories. Weight (to the nearest 0.1kg) and body fat % (to the nearest 0.1kg) was measured using Tanita 611 scales (Tanita, West Drayton, UK). Height was measured to the nearest 0.1cm using a stadiometer. Waist circumference was measured by trained staff using a non-stretching measuring tape over the tops of the iliac crests. Smoking was self-reported. Postcode was used to calculate the Index of Multiple Deprivation (IMD), which is a deprivation score for small areas in England based on a combination of domains encompassing economic, social and housing factors, and enabled ranking of areas according to their specific level of deprivation [37]. Physical activity was assessed with the short-format International Physical Activity Questionnaire (IPAQ), which assesses moderate to vigorous intensity activities carried out for greater than 10 minutes within the previous 7 days [38]and was expressed as metabolic equivalents (METs). Blood biochemistry (HbA1c, glucose and lipids) was measured by the University Hospitals of Leicester, Clinical Pathology Services. Glucose tolerance was assessed using a 75g oral glucose tolerance test (OGTT) and participants were categorised as having normal glucose tolerance (NGT) and impaired glucose regulation (IGR) according to World Health Organisation criteria (WHO) [39].

### 2.3 Biomarker measurement

Prior to screening, participants were instructed to fast for 12 hours before the study visit. Venous blood was collected in EDTA tubes, centrifuged at 1500 *g* for 10 minutes to produce plasma, this was then frozen at −80°C for subsequent measurement of research biomarkers. Quantitative analysis of plasma for human CRP, ApoA1 and ApoB was carried out on the ABX Pentra clinical chemistry analyser and latex-enhanced immunoturbidimetric assay (Horiba medical, Northampton, UK). TNF-α and IL-6 were analysed using the Quantikine high sensitivity ELISA for human TNF-alpha /TNFSF1A and human IL-6 kit, (R&D Systems, UK). Leptin and resistin was assayed using the Mediagnost human enzyme-immunoassay ELISA kits (Reutlingen, Germany). Human adiponectin, insulin and 8-Iso prostaglandin F2 (8-Iso-PG F2) were assayed fluormetrically using the AutoDELFIA 1235 automatic immunoassay systems (Perkin Elma, Buckinghamshire UK). All samples were analysed in duplicate and with all duplicate samples having a CV% of ≤20%. All research biomarker assessments were carried out by University of Leicester Scientists in conjunction with Unilever personnel at the Unilever Corporate Research Laboratory, Bedford UK.

### 2.4 Statistical analysis

Demographic variables were presented as mean (SD) or n (%) for continuous and categorical variables respectively. Differences between the groups with and without depressive symptoms were estimated using chi-squared tests for categorical variables and t-tests for continuous variables. Linear regression models were fitted for continuous response variables and logistic regression models were fitted for binary response variables. All models included a binary depression case status variable, as well as additional known confounding variables. These covariates were selected *a priori* on the basis of previously reported associations with depression, diabetes or cardiovascular disease. The covariates included were: age, sex, ethnicity, BMI, IMD, smoking status and physical activity. Adjusted means were calculated from linear regression models for groups with and without depressive symptoms, whilst odds ratios were calculated from logistic regression models for the group without depressive symptoms. Models were fitted for the whole cohort and subsequently stratified by gender. Additionally, the interaction between depression and gender was assessed by including an interaction term in the model. Additionally, the analysis was repeated substituting BMI with waist circumference. Data was analysed using Stata V12.1. No adjustments were made for multiple testing. P values of ≤0.05 were considered statistically significant.

## 3. Results

### 3.1 Study population

The descriptive statistics of the whole cohort are summarised in Table 1. Within the current study, 25% of the cohort (31.4% of women and 19.4% of men) were categorised as having depressive symptoms. The cohort with depressive symptoms were less physically active, 2807.5(3194.7) vs. 3524 (+/-3685.7) METS/week, p = 0.029 and were more likely to be current smokers (p = 0.047) and this is in line with previous findings [8, 40]. There was no significant difference in the prevalence of NGT, IGR or screen-detected T2DM within each cohort. A data table outlining the characteristics of the excluded subjects is outlined in S1 Table.

**Table 1:** Descriptive characteristics of the study population.

Variable	Depressive Symptoms	No depressive symptoms	All	P-value^a^
Sex				
Male	67 (42.1)	279 (58.1)	346 (54.2)	*<0*.*001*
Female	92 (57.9)	201 (41.9)	293 (45.9)	
Age, years	56.5 (10.6)	59.5 (9.8)	58.7 (10.1)	*0*.*001*
Ethnicity				
White European	121 (76.1)	373 (77.7)	494 (77.3)	
South Asian	36 (22.6)	104 (21.7)	140 (21.9)	0.704
Other	2 (1.3)	3 (0.6)	5 (0.8)	
Glycaemia status				
Normal glucose tolerance	78 (49.1)	252 (52.5)	330 (51.6)	0.286
Impaired glucose regulation	65 (40.9)	164 (34.2)	229 (35.8)	
Type 2 diabetes	15 (9.4)	63 (13.1)	78 (12.2)	
Missing	1 (0.6)	1 (0.2)	2 (0.3)	
BMI, kg/m^2^	30.1 (5.2)	29.5 (4.5)	29.6 (4.7)	0.185
Waist circumference, cm	99.1 (13.6)	98.5 (12.3)	98.7 (12.6)	0.636
Smoking status				
Non-smoker	87 (54.7)	258 (53.8)	345 (54.0)	*0*.*047*
Current smoker	28 (17.6)	53 (11.0)	81 (12.7)	
Ex-smoker	44 (27.7)	169 (35.2)	213 (33.3)	
IMD Score	20.2 (12.4)	18.7 (12.3)	19.1 (12.3)	0.208
Total METs/week	2807.5 (3194.7)	3524.0 (3685.7)	3345.7 (3580.8)	*0*.*029*
**Total**	**159 (100.0)**	**480 (100.0)**	**639 (100.0)**	

All values are n (%) or mean (sd). Abbreviations: BMI, Body Mass Index; IMD, Index of Multiple Deprivation; SD, Standard Deviation. Percentages are in parenthesis.

^a^ P-values test for a difference between the groups with and without depressive symptoms, and were estimated using chi-squared tests for categorical variables and t-tests for continuous variables. Missing values for continuous variables: 1 Waist circumference.

### 3.2 Study populations stratified by gender

Table 2 outlines the descriptive characteristics of the cohort stratified by gender. Men with depressive symptoms were younger (p = 0.001) and had 7.8% lower body fat (p = 0.015) than men without. Women with depressive symptoms were marginally younger (p = 0.061), were 2.5 times more likely to be current smokers (p = 0.029) and had a 5.3% larger waist circumference (p = 0.003) than women without.

**Table 2:** Descriptive characteristics of the study population stratified by gender.

	Men			Women		
Variable	Depressive symptoms (n = 67)	No Depressive symptoms (n = 279)	P-value^a^	Depressive symptoms (n = 92)	No Depressive s symptoms (n(n = 201)	P-value^a^
Age, years	54.4 (10.2)	58.8 (9.9)	*0*.*001*	58.0 (10.7)	60.4 (9.7)	0.061
Ethnicity						
White European	51 (76.1)	208 (74.6)	0.791	70 (76.1)	165 (82.1)	
South Asian	16 (23.9)	71 (25.5)		20 (21.7)	33 (16.4)	0.486
Other	0 (0.0)	0 (0.0)		2 (2.2)	3 (1.5)	
Glycaemia status						
Normal glucose tolerance	34 (50.8)	142 (50.9)	0.923	44 (47.8)	110 (54.7)	0.172
Impaired glucose regulation	24 (35.8)	93 (33.3)		41 (44.6)	71 (35.3)	
Type 2 diabetes	9 (13.4)	43 (15.4)		6 (6.5)	20 (10.0)	
Missing	0 (0.0)	1 (0.4)		1 (1.1)	0 (0.0)	
BMI, kg/m^2^	28.7 (4.4)	29.3 (3.9)	0.284	31.0 (5.6)	29.7 (5.3)	0.057
Waist circumference, cm	101.3 (11.5)	102.8 (10.9)	0.312	97.5 (14.8)	92.6 (11.8)	*0*.*003*
Body fat %	28.1 (7.0)	30.3 (6.4)	*0*.*015*	41.3 (7.2)	40.1 (5.9)	0.129
Smoking status						
Non-smoker	31 (46.3)	122 (43.7)	0.415	56 (60.9)	136 (67.7)	*0*.*029*
Current smoker	13 (19.4)	40 (14.3)		15 (16.3)	13 (6.5)	
Ex-smoker	23 (34.3)	117 (41.9)		21 (22.8)	52 (25.9)	
IMD Score	19.1 (11.9)	18.4 (11.5)	0.639	20.9 (12.8)	19.2 (13.3)	0.304
Total METs/week	3528.6 (3871.4)	4091 (4221.1)	0.320	2282.3 (2487.7)	2736.7 (2587.7)	0.159
**Total**	**67 (100.0)**	**279 (100.0)**		**92 (100.0)**	**201 (100.0)**	

All values are n (%) or mean (sd). Abbreviations: BMI, Body Mass Index; IMD, Index of Multiple Deprivation; SD, Standard Deviation. Percentages are in parenthesis.

^a^ P-values test for a difference between **participants with and without depressive symptoms**, and were estimated using chi-squared tests for categorical variables and t-tests for continuous variables.

Missing values for continuous variables: 1 Waist circumference; 8 Body fat %.

### 3.3 Insulin resistance

HOMA IR was not significantly higher in the group with depressive symptoms when analysing the entire mixed gender cohort (Table 3). However, stratification for gender showed that HOMA IR (and fasting insulin) was 28.7% higher in women with depressive symptoms vs. women without (p = 0.026, Table 4). To assess potential mediators of this difference, the covariate BMI was substituted with waist circumference as this was shown to be significantly higher in women with depressive symptoms within this cohort, whilst BMI and body fat % were highly comparable in women with and without depressive symptoms (Table 2). Inclusion of waist circumference as a covariate attenuated the significant association between depressive symptoms and HOMA IR (p = 0.095, S2 Table), suggesting that the higher HOMA IR levels were, in part, mediated through higher levels of central obesity, however unadjusted regression analysis between HOMA IR and waist circumference showed only a moderate association R^2^ = 0.188. Despite the depression-linked elevation in IR in women, HOMA IR levels were higher in men compared with women (p = 0.005)

**Table 3:** A comparison of participants with and without depressive symptoms, adjusted data.

	Adjusted mean (95% CI)^a^		
Variable	Depressive symptoms (n = 159)	No depressive symptoms (n = 480)	P-value
Resistin ng/ml	5.85 (5.01, 6.69)	5.68 (5.19, 6.16)	0.858
Apo B ng/ml	1.11 (1.06, 1.16)	1.15 (1.12, 1.18)	0.273
Apo A ng/ml	1.58 (1.52, 1.63)	1.54 (1.51, 1.57)	0.388
HDL cholesterol mmol/L	1.36 (1.31, 1.41)	1.33 (1.30, 1.36)	0.600
LDL cholesterol mmol/L	3.46 (3.30, 3.61)	3.61 (3.52, 3.70)	0.119
IL-6pg/ml	2.70 (2.38, 3.02)	2.51 (2.33, 2.70)	0.328
TNF-alpha ng/ml	1.89 (1.66, 2.12)	1.69 (1.56, 1.83)	0.080
Adiponectin μg/ml	17.5 (16.0, 19.1)	16.7 (15.80, 17.6)	0.377
Insulin mIU/L	10.77 (9.43, 12.12)	9.52 (8.74, 10.29)	0.224
Log-Leptin ng/ml	2.95 (2.87, 3.03)	2.62 (2.58, 2.67)	*0*.*013*
CRP mg/L	5.07 (4.29, 5.85)	3.69 (3.24, 4.14)	*0*.*041*
PGF2 pg/ml	2.58 (2.23, 2.93)	2.72 (2.52, 2.92)	0.408
Fasting glucose mmol/L	5.62 (5.40, 5.84)	5.64 (5.52, 5.77)	0.949
2 hour glucose mmol/L	7.62 (7.05, 8.19)	7.64 (7.31, 7.96)	0.896
HbA1c %	5.88 (5.75, 6.01)	5.93 (5.86, 6.01)	0.384
HOMA IR	2.78 (2.34, 3.22)	2.53 (2.27, 2.78)	0.470

^a^Adjusted for: age, sex, ethnicity, body mass index, Index of Multiple Deprivation, International Physical Activity Questionnaire score and smoking status.

**Table 4:** A comparison of participants with and without depressive symptoms stratified by sex, adjusted data.

	Adjusted Mean (95% CI)^a^					
	Men			Women		
Variable	Depressive symptoms (n = 67)	No depressive symptoms (n = 279)	P-value	Depressive symptoms (n = 92)	No depressive symptoms (n = 201)	P-value
Resistin ng/ml	5.50 (4.63, 6.38)	5.34 (4.91, 5.76)	0.687	6.06 (4.63, 7.49)	6.10 (5.04, 7.15)	0.879
Apo B ng/ml	1.11 (1.03, 1.19)	1.16 (1.12, 1.20)	0.421	1.08 (1.01, 1.14)	1.10 (1.05, 1.15)	0.454
Apo A ng/ml	1.47 (1.39, 1.55)	1.48 (1.44, 1.52)	0.845	1.64 (1.57, 1.71)	1.61 (1.55, 1.66)	0.240
HDL cholesterol mmol/L	1.20 (1.13, 1.28)	1.20 (1.16, 1.24)	0.418	1.46 (1.39, 1.54)	1.51 (1.45, 1.56)	0.984
LDL cholesterol mmol/L	3.38 (3.13, 3.62)	3.57 (3.45, 3.70)	0.138	3.47 (3.26, 3.67)	3.62 (3.48, 3.77)	0.345
IL-6 pg/ml	2.85 (2.34, 3.36)	2.57 (2.31, 2.82)	0.323	2.60 (2.17, 3.03)	2.42 (2.12, 2.72)	0.672
TNF-alpha ng/ml	1.93 (1.50, 2.37)	1.83 (1.61, 2.04)	0.679	1.86 (1.62, 2.09)	1.52 (1.36, 1.69)	*0*.*015*
Adiponectin μg/ml	13.0 (11.1, 14.9)	13.1 (12.2, 14.1)	0.401	20.8 (18.3, 23.3)	21.3 (19.5, 23.0)	0.499
Insulin mIU/L	12.13 (9.49, 14.76)	10.70 (9.40, 12.00)	0.714	10.13 (9.04, 11.23)	7.87 (7.09, 8.64)	*0*.*011*
Log-Leptin ng/ml	2.37 (2.24, 2.50)	2.21 (2.15, 2.28)	0.189	3.40 (3.29, 3.51)	3.19 (3.11, 3.26)	*0*.*010*
CRP mg/L	4.53 (3.47, 5.60)	3.07 (2.55, 3.59)	*0*.*031*	5.39 (4.18, 6.60)	4.50 (3.64, 5.34)	0.427
PGF2 pg/ml	2.44 (1.90, 2.97)	2.64 (2.37, 2.90)	0.447	2.62 (2.13, 3.11)	2.79 (2.44, 3.14)	0.647
Fasting glucose mmol/L	5.92 (5.51, 6.33)	5.83 (5.63, 6.03)	0.954	5.39 (5.19, 5.60)	5.38 (5.24, 5.53)	0.871
2 hour glucose mmol/L	8.33 (7.32, 9.33)	7.84 (7.34, 8.33)	0.493	7.19 (6.54, 7.83)	7.41 (6.95, 7.86)	0.619
HbA1c %	5.98 (5.74, 6.22)	5.96 (5.84, 6.08)	0.792	5.81 (5.67, 5.94)	5.89 (5.79, 5.98)	0.331
HOMA IR	3.30 (2.42, 4.19)	2.95 (2.51, 3.38)	0.902	2.51 (2.19, 2.83)	1.95 (1.72, 2.17)	*0*.*026*

^a^Adjusted for: age, ethnicity, body mass index, Index of Multiple Deprivation,International Physical Activity Questionnaire score and smoking status.

### 3.4 Leptin

Levels of leptin were examined as continuous, log-transformed values and were higher with depressive symptoms (p = <0.013, Table 3). Stratification based on gender revealed that log-leptin was 6.6% higher in women with depressive symptoms compared with women without (p = 0.01, Table 4). Mediation analysis revealed that this association was in part mediated through central obesity as substituting BMI with waist circumference attenuated the association (p = 0.112, S2 Table). Indeed, unadjusted regression analysis between log-leptin and waist circumference showed only a moderate association R^2^ = 0.368. Leptin was significantly higher in women vs. men (p = <0.001).

### 3.5 Inflammation

#### 3.5.1TNF-α

When analysing the entire mixed gender cohort, TNF-α was not significantly higher in the group with depressive symptoms (Table 3), however stratification for gender showed that TNF-α was 22.3% higher in women with depressive symptoms vs. women without (p = 0.015, Table 4). Substitution of BMI with waist circumference did not attenuate this association (p = 0.016, S2 Table) and this is reflected by the finding that regression analysis of waist circumference and TNF-α showed a negligible association, R^2^ = 0.002. Further analysis showed that TNF-α levels in men and women were comparable (p = 0.486).

#### 3.5.2 CRP

CRP was also higher with depressive symptoms when assessing the entire cohort (p = 0.041, Table 3). Stratification for gender showed that CRP was 49% higher in men with depressive symptoms vs. men without (p = 0.031, Table 4) whilst no significant relationship was found in women. Further analysis showed that mean CRP levels were higher in women compared with men (p≤0.001).

### 3.6 The interaction of gender

Interaction analysis revealed that the association each biomarker and depressive symptoms did not significantly differ with gender within this cohort.

## 4. Discussion

The aim of the present cross-sectional analysis was to assess whether depressive symptoms were associated with an increase in markers for T2DM, CVD and inflammation within a healthy population. Additionally we hypothesized that any metabolic disturbances would differ with gender. Analysis of the mixed gender cohort showed that depressive symptoms were positively associated higher leptin and CRP levels (plus a higher likelihood of smoking and lower physical activity levels) and these findings agree with previous studies [8, 10, 13]. Stratification of the data for gender appeared to demonstrate that men and women show a different metabolic profile with depression. Depression in women was associated with a larger waist circumference and higher levels of adiposity/T2DM related markers specifically HOMA IR, leptin and TNF-α compared with women without depression. Conversely, depression in men was associated with lower body fat and 49% higher CRP levels compared to men without depression. However, interaction analysis revealed that the metabolic disturbances associated with depressive symptoms did not significantly differ with gender. This may reflect the relatively low number of participants included in the current analysis and we feel that further work including a larger cohort may successfully highlight gender-differences associated with depressive symptoms.

### 4.1 Elevated adiposity, IR, leptin and tnf-α in women

Systematic reviews have found a significant relationship between adiposity and depression [41, 42]; with more in depth studies revealing that the strongest association is found between depression and central obesity levels and our data agrees with these findings [43, 44]. One proposed mechanism for the higher central obesity with depression is thought to be an increased intake of calorie dense foods which preferentially cause increases in omental fat [45, 46]. Within the present study, HOMA IR and leptin were significantly higher in women with depressive symptoms, however this association was attenuated by accounting for waist circumference and this is in line with previous studies [13, 28, 47, 48, 49]. However, whilst omental fat clearly has a role in mediating the link between depression and IR and leptin levels, within the present study regression analysis of waist circumference vs. HOMA IR and leptin showed that the associations were relatively modest (R^2^ = 0.188 and 0.368), suggesting that other pathways, such as depression-linked excitation of the HPA axis, may also play a significant role in inducing metabolic derangement.

The above discussion suggests that depression leads to increases IR and leptin, however, due the cross-sectional design of this study the directionality of the association can not be proven. Some studies have shown that high baseline levels (or administration) of leptin and TNF-α lead to increased rates of *de novo* depression/onset depression-like symptoms [28, 50]. Therefore, it may be the case that an increase in central obesity contributes to an up-regulation of leptin and inflammatory factors which in turn leads to the onset depressive symptoms, whilst elevated IR seen with depression is purely a reflection of higher central adiposity.

### 4.2 Clinical relevance of elevated IR, leptin and TNF-α

The finding that HOMA IR was 28.7% higher with depressive symptoms suggests an increased risk of developing T2DM. Additionally, a prospective study by Facchini et al (2001) reported that IR was an independent predictor of cardiovascular disease, stroke, hypertension and cancer [51]. The elevated levels of TNF-α seen within the female cohort with depressive symptoms also represents an adverse metabolic profile as it is well recognised that elevations in TNF-α are associated with impaired glucose tolerance and IR, indeed, short-term TNF-α infusions have been shown to induce insulin resistance in healthy lean subjects [52]. Additionally, elevations in proinflammatory cytokines have been shown to lead to a decrease in serotonin production (reviewed by [53]) and therefore may perpetuate depression. Elevated leptin levels have been implicated in the development of hypertension in human and animal models [54, 55]. Moreover, hyperleptinemia has been associated with subclinical markers of atherosclerosis such as coronary calcifications and carotid artery intima-media thickness, indeed leptin inhibition has been postulated to be a promising strategy to slow atherogenesis in hyperleptinemic individuals [24].

### 4.3 Elevated CRP in men

Previous reports have shown that CRP is significantly elevated with depression, particularly in men and the results of this study agree with these findings [56, 57]. Again the directionality between high CRP and depressive symptoms cannot be inferred from these results. A longitudinal study (in women) carried out by Pasco and colleagues reported a positive association between high baseline CRP levels and increased risk of de novo major depression, in those with no previous history of depression [22]. The findings of Pasco et al are supported by the finding that proinflammatory cytokines activate the enzyme indoleamine-2,3-dioxygenase, an activation which leads to decreased production of serotonin (reviewed by [53]).

In line with women, men also showed a trend towards higher IR, leptin and TNF-α with depressive symptoms but within this cohort this did not reach significance (Table 4). This fact is interesting in light of the lower body fat % (and marginally lower BMI and waist circumference) shown in men with depressive symptoms. This finding suggests adiposity independent pathways may be driving metabolic derangement in men.

### 4.4 Clinical relevance of elevated CRP

CRP is recognised as an accurate predictor of future cardiovascular events with, 0-1mg/L predicting<6% risk of a cardiovascular event in the next 10 years (low risk), 1-3mg/L predicting a 6–20% risk (average risk) and >3-10mg predicting >20% risk (high risk) [58]. The male cohort without depression showed a mean CRP value of 3.07mg/L (95% CI 2.55,3.59) whilst the depressed cohort showed a mean value of 4.53mg/L (95% CI 3.47,5.60) and although both cohorts largely fall within the high risk category, the cohort with depressive symptoms are clearly at higher risk. Interestingly, there is evidence that systemic inflammation falls following remission of depression [59], additionally Elovainio and colleagues (2009) found that the more recent the diagnosed depression or depressive episode was, the stronger the relationship between depression and high CRP [57]. Therefore, amelioration of depressive episodes through psychotherapy/medication should have a significant impact on future metabolic morbidity.

### 4.5 Potential treatment of depression

As already outlined, due to the cross-sectional design of the present study it is possible that increases in inflammation and markers of T2DM and CVD precede the onset of symptoms of depression. This data potentially suggest that, for example, depression in women may respond to therapies aimed to reduce omental fat (diet and exercise) and TNF blockade medications [60]. Conversely depression in men is perhaps less likely to respond to weight-reducing therapies but may respond well to anti-depressant treatments that have been shown to be efficacious in individuals with high baseline CRP levels [60,61]. Having the ability match patients with treatment based on a variety of variables, including gender, and should help achieve the goal of highly personalised treatment, however further work on larger cohorts is required to ascertain whether true gender differences exist.

### 4.6 Strengths and limitations of the study

The main strengths of this study were that the data was adjusted for a wide range of potential confounders. Additionally, this study assessed a broad range of clinical and research biomarkers associated with T2DM, CVD and inflammation. However, we feel that the due to the relatively low number of participants included in this study (n = 639) we were unable to prove a significant difference between depressive symptoms and metabolic status in men and women. Additionally, this is a secondary analysis of a study designed to test a different primary hypothesis and therefore some measurement bias and residual confounding is likely. The temporal relationship between depressive symptoms and metabolic disturbances could not be established due to the cross-sectional design of the study. History of depression (depression duration and severity) and antidepressant/psychiatric medications were not taken into account, additionally, clinical diagnosis of depression was not confirmed in accordance with the Diagnostic and Statistical Manual of Mental Disorders (DSM-5)[62]. One further point to discuss is that Awata and colleagues have shown that the WHO (5) with a cut off of ≤13 has 100% sensitivity (i.e. 100% of depressed people were captured using this cut off) and 78% specificity compared with the clinical gold standard diagnosis of depression [35]. It could be argued that this cut-off categorises some individuals without depressive symptoms within the depressive symptoms group, therefore ‘diluting the data’. Therefore it is likely that further studies including a more stringent depression screen could increase the significance of the present findings.

## 5. Conclusion

This study supports the theory that symptoms of depression are associated with metabolic disturbances. Whilst women with depressive symptoms had significantly higher risk factors associated with diabetes (central obesity levels, IR, leptin and TNF-α), depression in men was associated with higher levels of CRP which potentially suggests an increased risk of CVD. However, within the present cohort whilst each gender showed distinct trends, no significant gender difference was found. It is possible that further work assessing larger cohorts may reveal a significant gender difference between depressive symptoms and metabolic status.

## Supporting information

S1 Table A comparison of included and excluded participants. All values are n (%) or mean (sd). ^a^ P-values test for a difference between participants included and excluded from the main analysis, and were estimated using chi-squared tests for categorical variables and t-tests for continuous variables.
(DOC)

S2 Table A comparison of depressed vs. non depressed participants stratified by sex, adjusted data (inclusion of waist circumference as a covariate in the place of BMI). ^a^Adjusted for: age, ethnicity, waist circumference, Index of Multiple Deprivation, International Physical Activity Questionnaire score and smoking status.
(DOC)

## Abbreviations

BME: Black and Minority Ethnic
BMI: Body Mass Index
CI: Confidence Interva
CVD: Cardiovascular Disease
CRP: C-Reactive Protein
HbA1c: Glycated Haemoglobin
HDL: High Density Lipoprotein
HOMA: homeostatic model assessment
HR: Hazard Ratio
hsCRP: High Sensitivity C-reactive Protein
IGR: Impaired Glucose Regulation
IL: Interleukin
IMD: Index of Multiple Deprivation
INF: Interferon
IPAQ: International Physical Activity Questionnaire
LDL: Low Density Lipoprotein
MET: Metabolic Equivalent of Task
NGT: Normal Glucose Tolerance
PGF: Prostaglandin F
T2DM: Type 2 Diabetes Mellitus
TNF: Tumour Necrosis Factor and WHO (Five)
WHO (Five): Well-Being Index
