# Supplementary material for: The association between depressive symptoms and insulin resistance, inflammation and adiposity in men and women
Source: PLoS One. 2017 Nov 30;12(11):e0187448. doi: 10.1371/journal.pone.0187448 (PMC5708702; doi:10.1371/journal.pone.0187448)
Supplement: S1 Table — All values are n (%) or mean (sd). a P-values test for a difference between participants included and excluded from the main analysis, and were estimated using chi-squared tests for categorical variables and t-tests for continuous variables. (DOC) [file pone.0187448.s001.doc]

S1 Table

| Variable | Included in analysis | Excluded from analysis | All | P-value^a^ |
| --- | --- | --- | --- | --- |
| Sex |  |  |  |  |
| Male | 346 (54.2) | 189 (54.3) | 535 (54.2) | 0.961 |
| Female | 293 (45.9) | 159 (45.7) | 452 (45.8) |
| Age, years | 58.7 (10.1) | 61.3 (9.6) | 59.6 (10.0) | *<0.001* |
| Ethnicity |  |  |  |  |
| White European | 494 (77.3) | 127 (36.5) | 621 (62.9) |  |
| South Asian | 140 (21.9) | 138 (39.7) | 278 (28.2) | *<0.001* |
| Other | 5 (0.8) | 3 (0.9) | 8 (0.8) |
| Missing | 0 (0.0) | 80 (23.0) | 80 (8.1) |  |
| Glycaemia status |  |  |  |  |
| Normal glucose tolerance | 330 (51.6) | 114 (32.8) | 444 (45.0) | *<0.001* |
| Impaired glucose regulation | 229 (35.8) | 176 (50.6) | 405 (41.0) |
| Type 2 diabetes | 78 (12.2) | 56 (16.1) | 134 (13.6) |
| Missing | 2 (0.3) | 2 (0.6) | 4 (0.4) |
| BMI, kg/m^2^ | 29.6 (4.7) | 29.0 (4.8) | 29.4 (4.8) | 0.069 |
| Waist circumference, cm | 98.7 (12.6) | 96.9 (12.1) | 98.1 (12.5) | 0.052 |
| Smoking status |  |  |  |  |
| Non-smoker | 345 (54.0) | 170 (48.9) | 515 (52.2) | *<0.001* |
| Current smoker | 81 (12.7) | 22 (6.3) | 103 (10.4) |
| Ex-smoker | 213 (33.3) | 75 (21.6) | 288 (29.2) |
| Missing | 0 (0.0) | 81 (23.3) | 81 (8.2) |  |
| IMD Score | 19.1 (12.3) | 22.1 (13.3) | 20.1 (12.7) | *<0.001* |
| Total METs/week | 3345.7 (3580.8) | 2192.4 (3384.6) | 3195.1 (3574.8) | *0.003* |
| **Total** | **639 (100.0)** | **348 (100.0)** | **987 (100.0)** |  |
