# Supplementary material for: The association between depressive symptoms and insulin resistance, inflammation and adiposity in men and women
Source: PLoS One. 2017 Nov 30;12(11):e0187448. doi: 10.1371/journal.pone.0187448 (PMC5708702; doi:10.1371/journal.pone.0187448)
Supplement: S2 Table — aAdjusted for: age, ethnicity, waist circumference, Index of Multiple Deprivation, International Physical Activity Questionnaire score and smoking status. (DOC) [file pone.0187448.s002.doc]

S2 Table

|  | **Adjusted Mean (95% CI)**^a^ | | | | | |  |
| --- | --- | --- | --- | --- | --- | --- | --- |
|  | **Men** | |  | **Women** | |  |  |
| **Variable** | **Depressed**  **(n=66)** | **Not Depressed**  **(n=279)** | **P-value** | **Depressed**  **(n=92)** | **Not Depressed**  **(n=201)** | **P-value** |  |
| Resistin ng/ml | 5.64 (4.76, 6.52) | 5.44 (5.00, 5.88) | 0.665 | 6.00 (4.59, 7.40) | 5.99 (4.84, 7.15) | 0.904 |  |
| Apo B ng/ml | 1.13 (1.05, 1.21) | 1.17 (1.13, 1.21) | 0.442 | 1.07 (1.01, 1.13) | 1.09 (1.04, 1.14) | 0.541 |  |
| Apo A ng/ml | 1.50 (1.42, 1.58) | 1.51 (1.46, 1.55) | 0.785 | 1.62 (1.55, 1.69) | 1.56 (1.50, 1.62) | 0.100 |  |
| HDL cholesterol mmol/L | 1.25 (1.18, 1.33) | 1.24 (1.20, 1.28) | 0.351 | 1.43 (1.35, 1.50) | 1.44 (1.39, 1.50) | 0.695 |  |
| LDL cholesterol mmol/L | 3.42 (3.17, 3.66) | 3.61 (3.48, 3.74) | 0.142 | 3.45 (3.25, 3.66) | 3.61 (3.46, 3.77) | 0.324 |  |
| IL-6 pg/ml | 2.67 (2.16, 3.19) | 2.42 (2.15, 2.69) | 0.334 | 2.75 (2.32, 3.17) | 2.67 (2.35, 2.99) | 0.900 |  |
| TNF-alpha ng/ml | 1.94 (1.51, 2.38) | 1.82 (1.59, 2.05) | 0.625 | 1.86 (1.63, 2.10) | 1.53 (1.36, 1.71) | *0.016* |  |
| Adiponectin µg/ml | 13.5 (11.6, 15.4) | 13.3 (12.4, 14.3) | 0.323 | 19.8 (17.3, 22.2) | 19.4 (17.5, 21.3) | 0.251 |  |
| Insulin mIU/L | 9.80 (7.14, 12.46) | 9.21 (7.82, 10.61) | 0.964 | 11.04 (9.92, 12.17) | 9.25 (8.40, 10.10) | *0.045* |  |
| Log-Leptin ng/ml | 2.10 (1.96, 2.23) | 2.02 (1.95, 2.09) | 0.469 | 3.55 (3.43, 3.66) | 3.42 (3.33, 3.50) | 0.112 |  |
| CRP mg/L | 4.01 (2.94, 5.08) | 2.70 (2.14, 3.26) | *0.044* | 5.85 (4.65, 7.05) | 5.30 (4.40, 6.20) | 0.677 |  |
| PGF2 pg/ml | 2.59 (2.06, 3.13) | 2.74 (2.46, 3.02) | 0.530 | 2.59 (2.10, 3.08) | 2.71 (2.34, 3.09) | 0.763 |  |
| Fasting glucose mmol/L | 5.79 (5.37, 6.20) | 5.72 (5.50, 5.93) | 0.969 | 5.49 (5.28, 5.69) | 5.53 (5.38, 5.69) | 0.831 |  |
| 2 hour glucose mmol/L | 8.15 (7.14, 9.15) | 7.66 (7.13, 8.18) | 0.469 | 7.30 (6.66, 7.94) | 7.66 (6.18, 8.14) | 0.393 |  |
| HbA1c % | 5.90 (5.67, 6.14) | 5.88 (5.76, 6.01) | 0.829 | 5.86 (5.73, 5.99) | 5.97 (5.87, 6.07) | 0.194 |  |
| HOMA IR | 2.59 (1.70, 3.48) | 2.47 (2.00, 2.94) | 0.894 | 2.79 (2.46, 3.11) | 2.37 (2.12, 2.61) | 0.095 |  |
